# Postoperative Radiation Therapy for Non-Small Cell Lung Cancer and Thymic Malignancies

**DOI:** 10.3390/cancers4010307

**Published:** 2012-03-14

**Authors:** Daniel R. Gomez, Ritsuko Komaki

**Affiliations:** Department of Radiation Oncology, The University of Texas MD Anderson Cancer Center, 1840 Old Spanish Trail, Houston, TX 77054, USA; E-Mail: rkomaki@mdanderson.org

**Keywords:** postoperative radiation therapy, superior sulcus tumors, thymoma

## Abstract

For many thoracic malignancies, surgery, when feasible, is the preferred upfront modality for local control. However, adjuvant radiation plays an important role in minimizing the risk of locoregional recurrence. Tumors in the thoracic category include certain subgroups of non-small cell lung cancer (NSCLC) as well as thymic malignancies. The indications, radiation doses, and treatment fields vary amongst subtypes of thoracic tumors, as does the level of data supporting the use of radiation. For example, in the setting of NSCLC, postoperative radiation is typically reserved for close/positive margins or N2/N3 disease, although such diseases as superior sulcus tumors present unique cases in which the role of neoadjuvant *vs.* adjuvant treatment is still being elucidated. In contrast, for thymic malignancies, postoperative radiation therapy is often used for initially resected Masaoka stage III or higher disease, with its use for stage II disease remaining controversial. This review provides an overview of postoperative radiation therapy for thoracic tumors, with a separate focus on superior sulcus tumors and thymoma, including a discussion of acceptable radiation approaches and an assessment of the current controversies involved in its use.

## 1. Introduction

The tenets of postoperative radiation therapy (PORT) in the setting of locally advanced non-small cell lung cancer (NSCLC) have evolved significantly over the past several decades. Not only have the indications become clearer, with increasing outcome data regarding survival outcomes and improved characterization of patients who receive the greatest benefit from this treatment, but also from a standpoint of imaging and radiation delivery, advancing technology now allows physicians to better target the specific region of interest while avoiding surrounding critical structures. With this innovation, there remains debate as to the appropriate field that should be included in the postoperative setting, and whether traditional larger fields can be reduced to decrease toxicity without compromising locoregional control.

In contrast, in the setting of thymic malignancies, the indications for PORT are still unclear to a great extent. Given the rarity of this disease, data regarding outcomes in the postoperative setting are limited to single-institution retrospective trials and analysis of large, population-based datasets that are heterogeneous in terms of treatment paradigms and often lack key variables that could aid the treating physician in deciding the appropriate approach for an individual patient. Furthermore, this body of data has been conflicting in regards to the true benefit of PORT, particularly in Masaoka-Koga stage II and III disease. However, the postoperative treatment of thymic malignancies has also advanced through the development and implementation of modern imaging and radiation techniques, particularly with the location of these tumors in the anterior mediastinum.

This review provides an overview of the indications and techniques for PORT in the setting of NSCLC. Special consideration will be given to superior sulcus tumors, which have historically been approached uniquely. Next, the review will discuss the use of PORT to treat patients with thymic malignancies, with a presentation of the data supporting and advocating the omittance of PORT in stage II and III disease. Radiation delivery will be discussed in depth as well, including the radiation dose, technique, and volumes in this clinical context.

## 2. Non-Small Cell Lung Cancer

### 2.1. Indications and Results—Surgical Margins

Multiple studies have considered the indications for PORT in patients with NSCLC, with general agreement that the use of PORT in the setting of positive surgical margins reduces the risk of locoregional recurrence (LRR) [1]. Positive margins have also been shown to be associated with worse overall survival [2,3]. For close surgical margins, the data are less clear regarding the utility of PORT, as is the definition of “close”. There is not a consensus in the published literature as to the margin at which LRR increases. For example, investigators from the University of Pittsburgh found that with sublobar resection, margins less than 1 cm were associated with a 15% risk of local recurrence, while recurrence rates were 7% when the distance was equal to or greater than this this threshold [4]. In contrast, researchers from Japan utilized a margin to tumor size ratio (M/T) of greater than 1 to predict cancer recurrence [5]. And investigators from the University of Mayo studied 3,936 consecutive pulmonary resections and found that as long as a negative resection margin is achieved, the extent of bronchial margin has no impact on disease-free survival [6]. Because the data is not consistent as to what margin constitutes an increased risk of local recurrence, at MD Anderson Cancer Center (MDACC), a strict distance threshold is not used as a criterion for PORT. Rather, the case is discussed with the operating surgeon to determine their assessment of the risk of residual disease based on the surgical findings. When patients are ultimately treated with PORT in this setting, the dose and technique utilized are similar to those in cases of positive margins, as described below.

### 2.2. Indications and Results—Stage and Nodal Status

Numerous randomized trials have investigated the utility of PORT in various stages of NSCLC. Selected studies are summarized in Table 1 [7,8,9,10,11,12]. It should be noted that the study by Douillard *et al*. was a retrospective analysis of a prospective trial assessing the efficacy of adjuvant vinorelbine (Navelbine) in the setting of locally advanced NSCLC. However, a clear benefit was shown for PORT in patients with N2 disease and in patients not receiving chemotherapy for N1 disease. Overall, it is evident from these trials that, whereas PORT appears to reduce the risk of locoregional recurrence, there is no apparent survival difference. As is evident in the trial by Dautzenberg *et al*., the lack of translation to a benefit in overall survival may be secondary to intercurrent disease as a result of the utilization of older radiation techniques.

**Table 1:** Selected clinical trials evaluating postoperative radiation therapy in the setting of non-small cell lung cancer.

Author/Name of Study	Year	No. of Patients	Inclusion Criteria	Survival Outcomes	Conclusions
Lung Cancer Study Group [7] (4)	1986	230 (110 with PORT)	Stage I–III	3 *vs*. 41% LRR with PORT (*p* = 0.001) 40 *vs*. 40% 5-year OS with PORT (*p* = NS)	PORT improved recurrence rate, no effect on OS.
Dautzenberg *et al*. [8] (5)	1999	728 (373 with PORT)	Stage I–III	5-year OS 30% *vs*. 43% with PORT (*p* = 0.002) Intercurrent death 31 *vs*. 8% with PORT	PORT detrimental in survival.
Douilliard *et al*. [9] (ANITA) (6)	2008	840 (232 with PORT)	I B–III A	MS–N1 Obs—50 *vs*. 26 months N1 Chemo—47 *vs*. 94 months N2 Obs—47 *vs*. 24 months N2 Chemo—23 *vs*. 13 months	Positive effect of PORT in pN2 disease and pN1 disease without chemotherapy.
Feng *et al*. [10] (7)	2000	366 (183 with PORT)	N1 and N2	13 *vs*. 33% thoracic failure with PORT (*p* < 0.01) 5-year OS 43 *vs*. 41% (*p* = NS)	PORT improved LRR but no impact on survival.
Mayer *et al*. [11] (8)	1997	155 (83 with PORT)	pT1–T3 pN0–N2	27 *vs*. 16% 5-year DFS with PORT (*p* = 0.07) 30 *vs*. 20% 5-year OS with PORT (*p* > 0.05)	PORT improved recurrence rate, no effect on OS.
Trodella *et al*. [12] (9)	2002	104 (51 with PORT)	Stage I	2 *vs*. 23% local recurrence with PORT. 71 *vs*. 60% 5-year DFS with PORT (*p* = 0.039). 67 *vs*. 58% OS with PORT (*p* = 0.048)	Improvement in local control with PORT, “promising trend” in 5-year OS and DFS.

PORT = postoperative radiation therapy; LRR = locoregional recurrence; OS = overall survival; DFS = disease-free survival.

Other than the clinical trials noted above, a widely cited retrospective study is a Surveillance, Epidemiology, and End Results (SEER) study that examined over 7,000 patients to determine the effect of PORT on overall survival. The authors found that the use of PORT on all patients did not significantly affect overall survival, yet there was a significant increase in overall survival in patients with N2 nodal disease. For patients with N0 and N1 nodal disease, PORT was associated with a significant decrease in survival [13].

Finally, the seminal study in this regard was the PORT Metaanalysis Trialists Group, which assessed nine trials of surgery with or without postoperative radiation. When the authors aggregated the data from these trials, they found that overall survival was 7% lower in patients with N0-N1 disease but that there was no difference in survival for patients with N2 disease [14]. This trial was subsequently updated in 2005, and again the authors did not find a benefit with PORT in the adjuvant setting [15]. It must be emphasized that the trials included in this analysis predominantly utilized less conformal radiation techniques and had a large proportion of patients with N0 status (approximately 25%) and that many patients did not undergo adequate staging.

Therefore, the current consensus recommendation, as given in the National Comprehensive Cancer Network (NCCN) guidelines, is to deliver PORT for N2 disease, because the consensus of the current published data indicates a benefit for this subgroup of patients. In the setting of N1 or N0 disease, PORT is considered in the setting of close or positive surgical margins [16]. However, the question of the utility of PORT in the setting of N2 disease will be addressed specifically in the Lung Adjuvant Radiotherapy (LungART) Trial, a phase III international study designed to compare PORT with no PORT in resected N2 NSCLC [17].

Other than the standard indications for PORT listed above, other investigations have attempted to elucidate patient subgroups in which PORT should be omitted or recommended. For example, Matsuguma *et al*. found that the impact of PORT was greater in those patients with multiple N2 nodal stations involved [18]. In another study, investigators from Japan found that an increased number of lymph nodes was associated with overall survival, with four lymph nodes being a threshold for poorer survival [19]. Finally, investigators from Vanderbilt University Medical Center assessed the impact of extranodal extension (ECE) in patients receiving PORT, and found that the survival benefit with PORT was present with negative ECE but not with positive ECE. In addition, ECE was associated with a lower locoregional survival rate [20]. The authors concluded that further investigation is indicated for PORT in this setting. While all of these factors (number of mediastinal stations involved, number of N1 lymph nodes involved, presence/absence of ECE) may play a role in deciding if PORT is indicated for an individual patient, they are not standard criteria. We thus recommend that patients with questionable indications for PORT be discussed in a multidisciplinary setting to weigh the risks and benefits of this approach.

### 2.3. Indications and Results—Radiation Therapy *vs.* Chemoradiation Postoperatively and Sequencing of Chemotherapy and Radiation Therapy

In 2000, the Eastern Cooperative Oncology Group published the results of a randomized trial of PORT *vs.* chemoradiation for patients with “completely resected” stage II-IIIA NSCLC. Patients were required to undergo either systematic sampling of mediastinal lymph nodes or complete dissection, the latter defined as removal of lymph nodes at levels 4, 7, and 10 if a right-sided thoracotomy was performed or at levels 5 or 6 and 7 for a left-sided thoracotomy. Patients were treated with radiation therapy to a total dose of 50.4 Gy given in 28 fractions with concurrent cisplatin (60 mg/m^2^) and etoposide (120 mg/m^2^). There was no difference in median survival or recurrence between the two groups, although both the extent of lymph node involvement (multiple *vs*. single) and the extent of lymph node dissection influenced recurrence-free and overall survival, favoring patients with single station involvement and more extensive lymph node dissections [21]. More recent data have suggested a benefit with adding chemotherapy to the postoperative setting. For instance, phase II trials [22,23] have demonstrated low rates of local failure (15%) and promising overall survival with chemoradiation (approximately 90% at 2 years), and a recent meta-analysis demonstrated a 4% overall survival benefit at 5 years with postoperative chemoradiation compared with radiation alone [24]. However, these data are not convincing enough to implement a wide-scale change in practice, and thus combined chemoradiation is not the standard in the postoperative setting unless there is a concern for residual disease.

Regarding the sequencing of chemotherapy *vs*. PORT in the adjuvant setting, given the benefit of adjuvant chemotherapy over surgery alone in multiple randomized trials the published data better supports initiating chemotherapy first to minimize the risk of distant metastasis. Indeed, this sequencing recommendation is reflected in the most recent American College of Radiology appropriateness criteria for postoperative adjuvant therapy in NSCLC [25]. However, for patients with close or positive surgical margins, initiating radiation or chemoradiation prior to systemic doses of chemotherapy may be appropriate, as also reflected in these guidelines. At MDACC, we discuss the operative and pathologic findings with the other members of the multidisciplinary team, and if it is thought that the patient is at a high risk for local recurrence then radiation therapy (with or without chemotherapy) will be performed prior to adjuvant chemotherapy.

### 2.4. Radiation Technique, Dose, and Fields

Patients are simulated for PORT in the supine position, strictly immobilized and with arms over the head to maximize the number of beam angles that are feasible. A four-dimensional simulation is recommended, to assess the extent of internal motion and for the purposes of treatment planning. We advocate conformal techniques to maximize sparing of surrounding critical structures. A dose-volume histogram should always be generated, and common dose constraints in the setting of a lobectomy include a mean lung dose of 20 Gy, less than 35% of the lung to receive greater than 20 Gy (V20 < 35%), mean esophagus dose of 34 Gy, maximum esophagus dose of 80 Gy, and less than 50% of the heart receiving 40 Gy (V40 < 50%) [26,27,28,29]. When a pneumonectomy is performed, we utilize constraints analogous to those that have been published in malignant pleural mesothelioma, that being a mean lung dose <8 Gy.

**Figure 1:**
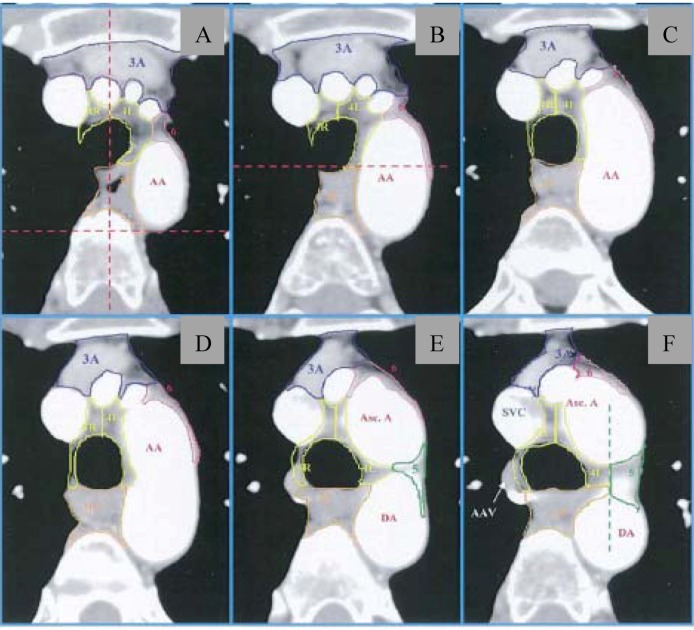
Thoracic atlas delineating lymph node stations from the University of Michigan (from Chapet *et al*. [23]). (**A**–**C**) Lymph node stations near level of sternal notch; (**D**) Superior aspect of aortic arch; (**E**) Level demonstrating posterior limit of 4R and 4L (red dotted line). (**F**) Level of left brachiocephalic vein, with delineation of 4L/3P and 5. AA = aortic arch; 3A = prevascular nodes; 3P = retrotracheal nodes; 4L/4R = lower paratracheal nodes; 5 = paraaortic nodes; 6 = paraaortic nodes.

Acceptable radiation doses are as follows (all in fraction sizes of 1.8–2.0 Gy): negative margins, 50–54 Gy; close or positive margins, 54–60 Gy; gross residual disease, 60–70 Gy. Historically, with conventional techniques, large radiation fields have been utilized for PORT. A representative field encompassed the ipsilateral hilum and bilateral mediastinum, extending superiorly/inferiorly from the thoracic inlet to 5 cm below the carina. However, with the advent of conformal techniques, nodal levels can be more accurately delineated, thus allowing for reduced treatment volumes and thus a decreased risk of toxicity. Utilizing CT-based planning, investigators from the University of Michigan published a thoracic atlas that defined the CT boundaries of lymph node stations 1–2, 3, 4, 5, 6, 7, 8, and 10–11 [30]. Representative images from this atlas are shown in Figure 1. And the most recent edition of the American Joint Committee on Cancer Staging System (7th ed.) provides a detailed definition of lymph node stations by contrasting the systems by the Japan Lung Cancer Society Map, the Mountain-Dresler modification of the American Thoracic Society (MD-ATS) lymph node map, and the International Association of the Study of Lung Cancer (IASLC) anatomical definition. The interested reader is referred to this manual for a comparison of these systems [31]. However, per this staging system, the IASLC lymph node map is now the recommended means of delineating lymph node involvement.

Regarding treatment fields, the superior-inferior location of the primary tumor (*i.e*., upper lobe, lower lobe, *etc*.), does not typically affect our treatment field. Rather, the laterality of the primary tumor can influence the nodal stations that are covered. For example, the LungART trial has defined CT-based mediastinal volumes that are based on the sites of nodal positivity [32]. In the setting of positive level 7 lymph nodes and a right sided tumor, station 4R is included in the treatment field, while for a left-sided tumor station 4R is excluded from the treated volume while stations 4L and 5 are included.

At MDACC, we utilize CT-based planning and conformal techniques to cover at-risk nodal stations and the ipsilateral hilum/bronchial stump, akin to the fields advocated in the LungART trial. However, it must be emphasized that currently there is no consensus regarding the treatment volume, with some physicians utilizing comprehensive fields and others using smaller volumes, and there has never been a direct prospective comparison. Of note, a recent study attempted to estimate the magnitude of field size dependence on radiotherapy-induced mortality and tumor control after PORT by estimating the product of the probabilities of (residual local disease) × (sterilization of residual disease with PORT) × (absence of metastatic disease) from clinical trials in the literature; this study found that radiation-induced mortality did indeed offset much of the benefit of PORT [33]. However, it should be noted that the trials from this study utilized predominantly conventional techniques. With current techniques, larger fields are possible while sparing structures such as the heart, lungs, and esophagus. To this end, investigators from the University of Pennsylvania assessed the risk of intercurrent disease in patients receiving PORT with “modern” techniques, defined as computerized dosimetry, uniform linear accelerator therapy, and conventional fractionation. The authors found no increased risk of intercurrent deaths with these techniques [34]. Similarly, another analysis with the SEER database demonstrated that the risk of cardiac mortality after PORT has declined in recent years [35]. Finally, investigators from Canada compared three-dimensional with two-dimensional techniques in the setting of PORT and found that the use of three-dimensional techniques was associated with improved locoregional control, suggesting a benefit of improved localization [36]. Thus, it does appear that as conformality has increased in this setting, efficacy and toxicity profiles have improved, and thus a larger benefit may be realized with upcoming studies.

### 2.5. Postoperative Radiation Therapy—Superior Sulcus Tumors

Superior sulcus tumors represent a unique subset of NSCLC. These malignancies arise in the apex of the lung and often present with associated symptoms of nerve and bone involvement in that region. Specifically, patients often experience Pancoast’s syndrome (or a variation thereof), the description of which was first published in 1932 and includes shoulder pain, brachial plexopathy, and Horner’s syndrome [37]. In addition, due to the infiltrative nature of these tumors and proximity to critical structures, surgical resection is often difficult, with rates of gross total resection at high-volume medical centers being reported as 40–60% [38]. As a result, a preoperative chemoradiation approach has been utilized, as first reported in a cooperative study by the Southwestern Oncology Group [39,40]. With a preoperative regimen of 45 Gy with concurrent cisplatin and etoposide, the authors reported R0/R1 resection rates of 92% and a 5-year overall survival rate of 44%. Since this trial, several other investigators have reported on this approach with similar results [41,42,43,44].

One issue with this approach is that, particularly if “preoperative” radiation doses are utilized (*i.e*., 45 Gy), if surgical resection is not feasible after induction treatment then the added utility of 15–25 Gy of further radiation after a break for re-evaluation is likely modest. As a result, recent studies have evaluated the feasibility of using higher preoperative doses, with acceptable toxicity [42,45,46]. As an alternative approach, an upfront attempt at aggressive surgical resection *in select patients* would both provide quick relief of some symptoms of tumor infiltration, such as pain, and then allow the treating radiation oncologist to target the postoperative bed and high-risk regions on the basis of surgical findings. Investigators from Turkey retrospectively assessed an approach of immediate surgical resection compared with induction (chemo)radiation and found that, when a complete resection was achieved, the median survival was 24 months, with a 5-year overall survival rate of 37% [47]. MDACC has also published retrospective results of selective surgical resection in a multimodality setting based on the extent of tumor involvement, with 72% local control and a median overall survival time of 18 months [48].

In a recent publication, our center reported prospective results of a phase II study examining aggressive surgical resection followed by postoperative chemoradiation in patients with superior sulcus tumors. The postoperative radiation regimen included a hyperfractionated course of radiation therapy to 60–64.8 Gy in 1.2-Gy fractions, to minimize toxicity to the brachial plexus and spinal cord, and based on prior published studies with analogous regimens [49,50]. The chemotherapy regimen was cisplatin (50 mg/m^2^) and etoposide (50 mg/m^2^) for two cycles concurrent with radiation therapy. Table 2 demonstrates the comparative results of preoperative *vs.* postoperative radiation in superior sulcus tumors, as taken from Gomez *et al*. [51]. It is evident that the regimens are analogous with respect to adherence, treatment morbidity, resections, and survival outcomes. Thus, we recommend that all patients with this relatively rare but aggressive tumor undergo evaluation for upfront surgical resection at an experienced center and that, regardless of treatment approach, multimodality therapy be utilized to maximize the probability of local control.

**Table 2:** Comparison of preoperative with postoperative radiation in prospective trials (from Gomez *et al*. [43] and comparing this postoperative radiation trial with studies from the Southwest Oncology Group [31,32] and JCOG 9806 [36]).

Outcome Variables	Preoperative Concurrent Chemoradiation		Postoperative Concurrent Chemoradiation Current Study
	SWOG 94-16	JCOG 9806	
Adherence to treatment regimen	75% ^*^	76%	78%
Operative mortality rates	2.4%	3.5%	0
R0/R1 resection rates	92% ^**^	95%	100%
Locoregional control rates	85% ^***^	87% ^†^	76%
5-year overall survival rates	44%	56%	50%

^*^ Defined as percentage of patients undergoing induction chemoradiation followed by thoracotomy as a proportion of the total number of eligible patients; ^**^ Defined as percentage of patients in whom R0/R1 resection was achieved as a proportion of those patients who proceeded to surgery ^***^ Reported as local control. ^†^ Reported as initial site of failure; SWOG, Southwest Oncology Group; JCOG, Japan Clinical Oncology Group.

## 3. Thymoma

### 3.1. Indications and Results

In the setting of gross residual disease after surgery, PORT should be administered for definitive intent, with doses analogous to those for unresectable disease (outlined below), and concurrent chemotherapy should be considered. In the scenario of completely resected thymoma, PORT most often pertains to Masaoka stage II and III thymoma. Resected stage I disease does not require further treatment, and stage IV disease is often treated with systemic therapy alone or induction chemoradiation. Indeed, stage III thymoma is also often treated with preoperative chemotherapy and radiation, with good results [52]. However, a significant proportion of patients with stage III disease receive upfront surgery or have their disease upstaged at the time of resection.

Many studies have assessed the benefit of adjuvant radiation therapy for patients with thymoma; selected studies are outlined in Table 3 [53,54,55,56,57,58,59]. Due to the rarity of this disease, studies have been limited by the retrospective nature of analyses and by wide time ranges and hence various treatment techniques. However, it is apparent that there is no true consensus regarding the role of radiation therapy in stage II and III completely resected disease. At MDACC, postoperative treatment in this setting is individualized and based on criteria such the extent of disease on preoperative imaging, findings at the time of surgical resection, and patient comorbidities. The vast majority of these cases are discussed by a multidisciplinary tumor board to assess the risks and benefits of treatment in a specific patient. However, because the published literature seems to indicate a greater trend for a benefit in stage III disease compared with stage II, most of these patients ultimately receive PORT at MDACC.

### 3.2. Radiation Dose and Fields

In preparation for PORT in thymic malignancies, patients are simulated in a fashion similar to that outlined above for NSCLC, with arms over the head and with four-dimensional CT images acquired at the time of simulation for the purposes of treatment planning. Again, conformal techniques are encouraged to reduce the dose to surrounding critical structures. Retrospective data suggest a dose response in the postoperative setting for thymoma, particularly above a threshold dose of 45 Gy [60]. Therefore, acceptable postoperative radiotherapy doses are 45–54 Gy for complete resections and negative surgical margins, 54–60 Gy for positive surgical margins, and 60–70 Gy for gross residual disease.

**Figure 2:**
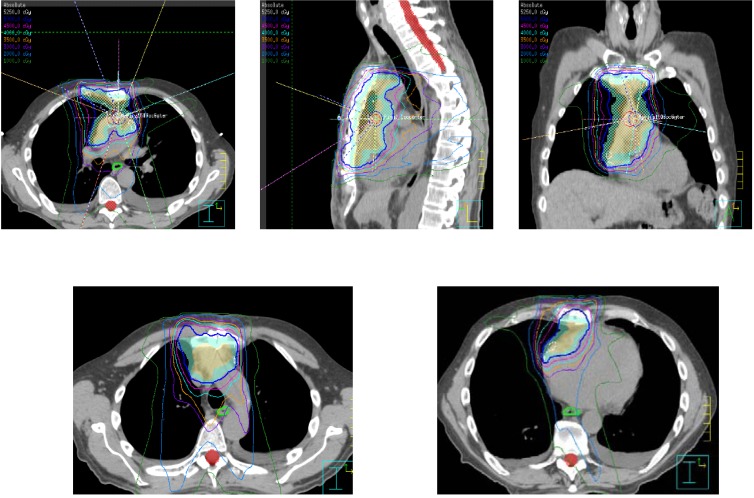
Representative postoperative treatment field in patient with locally advanced invasive thymoma.

Historically, the PORT radiation field for thymoma has encompassed elective mediastinal radiation. Furthermore, several investigators have examined the role of hemithoracic radiation, due to the propensity of this disease to spread to the pleura [61,62]. However, although toxicity has been shown to be acceptable with particular regimens, the dose that is feasible while limiting toxicity is often below the threshold that would be expected to be effective in controlling microscopic disease (<45 Gy), particularly with conventional techniques. Therefore, this technique remains experimental. At MDACC, we do not routinely treat elective regions of the mediastinum. The radiation field is typically designed to encompass the pretreatment superior-inferior extent of disease, while sparing regions such as lung parenchyma thought to be involved due to a “pushing”, rather than an infiltrative, border. Efforts are made to also include all surgical clips, although we discuss all cases with the treating surgeon to determine both the relationship of these clips to sites of involved disease as well as their assessment for any regions at particularly high risk that may require dose boosting, especially in the setting of positive surgical margins. Figure 2 demonstrates a typical postoperative field in this setting treated with intensity-modulated radiation therapy at MDACC.

**Table 3:** Selected clinical trials of postoperative radiation therapy for stage II and III thymoma.

Author/Name of Study	Year	No. of Patients	Masaoka Stage	Median (Range) RT Dose	Survival Outcomes	Conclusions
Berman *et al*. [53] (45)	2011	175	II	50.4 Gy (not reported)	Local recurrence rate 0% with PORT, 8% without PORT (*p* = 0.15)	PORT not beneficial in controlling local recurrence in Stage II disease.
Chang *et al*. [54] (46)	2011	76	II and III	50 Gy (43.2–66 Gy)	5-year DFS—98% with PORT, 80% without PORT 10-year DFS—93% with PORT, 70% without PORT (*p* = 0.043)	PORT beneficial in prolonging time to disease recurrence in Stage II and III thymoma.
Curran *et al*. [55] (47)	1988	57	II and III	50 Gy (32–60 Gy)	5-year DFS—100% with PORT, 45% with PORT (*p* = 0.12).	Nonsignificant trend towards improvement in DFS with PORT.
Forquer *et al*. [56] (48)	2010	901	Local and Regional Disease (SEER)	Not reported	Localized—5-year DSS 91% with PORT, 98% without PORT (*p* = 0.03). No difference in OS (*p* > 0.05) Regional—5-year OS 76% with PORT *vs*. 66% without PORT (*p* = 0.01). No difference in CSS.	PORT not beneficial in localized disease, may be beneficial in regional disease.
Kondo *et al*. [57] (49)	2003	1,320	II and III	Median not reported (<40–53.8 Gy)	II—Local recurrence 0% with PORT, 1.6% without PORT (*p* > 0.05) III—5.1% with PORT, 3.1% without PORT (*p* > 0.05)	PORT not beneficial in completely resected stage II or III disease.
Rena *et al*. [58] (50)	2007	197	II	Median not reported (45–54 Gy)	Five intrathoracic recurrences total, 3 with PORT and II without PORT (*p* = 0.432)	PORT not beneficial in stage II disease.
Utsumi *et al*. [59] (51)	2009	159	II and III	40 Gy (10–50 Gy)	II—100% DSS in all patients. III—88% DSS with PORT, 85% without PORT (*p* > 0.05).	PORT not beneficial in stage II or III disease.

RT = radiation therapy; PORT = postoperative radiation therapy; SEER = Surveillance, Epidemiology and End Results; DFS = disease-specific survival; OS = overall survival; DSS = disease-specific survival.

## 4. Conclusions

Postoperative radiation therapy plays a significant role in both NSCLC, including superior sulcus tumors, as well as thymic malignancies. In the setting of NSCLC, standard indications for PORT include positive surgical margins, gross residual malignancy, and N2 disease. For thymic malignancies, PORT is not used in Masaoka-Koga stage I disease and is controversial in resected stage II or III disease. Hemithoracic radiation in thymic malignancies is not well established and future studies will provide information on the utility of this technique with modern radiation modalities.
